# A146 THE EFFECTIVENESS OF USTEKINUMAB DOSE ESCALATION IN PATIENTS WITH ULCERATIVE COLITIS

**DOI:** 10.1093/jcag/gwab049.145

**Published:** 2022-02-21

**Authors:** L Albino, R Rosentreter, C Lu, J Siffledeen, L A Dieleman, C Ma, D C Baugmart, L Du, B Halloran, K Kroeker, F Peerani, K Wong

**Affiliations:** 1 University of Alberta Faculty of Medicine and Dentistry, Edmonton, BC, Canada; 2 Medicine, University of Calgary Cumming School of Medicine, Calgary, AB, Canada

## Abstract

**Background:**

Ustekinumab (UST), an IgG1 antibody that targets IL-12/23, is an effective and safe treatment option for patients with inflammatory bowel disease (IBD). Cohort studies have shown that dose escalation is an effective strategy for reinducing and maintaining remission in Crohn’s disease patients who do not respond or lose response to standard dosing of UST. There are currently no published studies evaluating effectiveness of UST dose escalation in ulcerative colitis (UC) patients.

**Aims:**

To assess the effectiveness of UST dose escalation in patients with moderate-to-severe UC who have not responded to or lost responsiveness to standard maintenance dosing (90mg SC every 8 weeks).

**Methods:**

A retrospective cohort study was conducted at three centers. Consecutive patients with moderate-to-severe UC initiated on ustekinumab were enrolled.

**Results:**

Data on 43 patients (26 males) are reported (to date, patients from 1 of 3 centres have been reviewed). Mean age was 40.2 years (±15.6). Mean duration of disease was 8.5 years (±5.8). Mean duration of follow up while on UST was 8.8 months (±7.2). In total, 28% (12) of patients underwent dose escalation: 8% (1) by way of IV reinduction, 58% (7) through interval shortening (every 4 weeks), and 33% (4) by both interval shortening and IV reinduction. Mean time to first dose escalation was 6.2 months (±4.1). Mean time to second dose escalation was 5.1 months (±1.2). Seven percent (3) of patients discontinued UST, with the mean timeframe being 5.3 months (±2.9). Three patients discontinued UST due to primary non-response with one proceeding onto surgery.

Time to normalization of CRP and FCP after initiation of UST is shown in Table 1.

**Conclusions:**

Preliminary data demonstrates that 28% of patients in this cohort required UST dose escalation, with 33% requiring a second dose escalation. Only 7% of patients discontinued UST at 9 months of follow up. Longer term follow up of this cohort would determine if dose escalation is an effective strategy to extend durability of ustekinumab.

Table 1. Normalization of CRP and FCP

**Funding Agencies:**

None

**Table:** 

	Non-Dose Escalated Patients	Dose Escalated Patients
Number of Patients	N=31	N=12
Elevated CRP (>8) Prior to UST Initiation	9/31 (29%)	5/12 (42%)
Normalized CRP Post-UST Initiation (Pre-Dose Escalation)	5/6 (83%)	1/5 (20%)
Mean Time to Normalization (Months)	1.6 (±1.0)	2
Elevated FCP (>100) Prior to UST Initiation	22/31 (76%)	8/12 (67%)
Normalized FCP Post-UST Initiation (Pre-Dose Escalation)	5/11 (45%)	2/8 (25%)
Mean Time to Normalization (Months)	5.4 (±4.3)	7 (±5)

